# Clustered Regularly Interspaced Short Palindromic Repeat/CRISPR-Associated Protein and Its Utility All at Sea: Status, Challenges, and Prospects

**DOI:** 10.3390/microorganisms12010118

**Published:** 2024-01-06

**Authors:** Jiashun Li, Shuaishuai Wu, Kaidian Zhang, Xueqiong Sun, Wenwen Lin, Cong Wang, Senjie Lin

**Affiliations:** 1 State Key Laboratory of Marine Environmental Science, College of Ocean and Earth Sciences, Xiamen University, Xiamen 361101, China; 2 State Key Laboratory of Marine Resource Utilization in the South China Sea, School of Marine Biology and Fisheries, Hainan University, Haikou 570203, China; 3 Department of Marine Sciences, University of Connecticut, Groton, CT 06340, USA

**Keywords:** CRISPR/Cas, genome editing technology, marine biology, functional genetic research, marine prokaryotic microbes, phytoplankton, zooplankton

## Abstract

Initially discovered over 35 years ago in the bacterium *Escherichia coli* as a defense system against invasion of viral (or other exogenous) DNA into the genome, CRISPR/Cas has ushered in a new era of functional genetics and served as a versatile genetic tool in all branches of life science. CRISPR/Cas has revolutionized the methodology of gene knockout with simplicity and rapidity, but it is also powerful for gene knock-in and gene modification. In the field of marine biology and ecology, this tool has been instrumental in the functional characterization of ‘dark’ genes and the documentation of the functional differentiation of gene paralogs. Powerful as it is, challenges exist that have hindered the advances in functional genetics in some important lineages. This review examines the status of applications of CRISPR/Cas in marine research and assesses the prospect of quickly expanding the deployment of this powerful tool to address the myriad fundamental marine biology and biological oceanography questions.

## 1. Genome Editing Technology

The Clustered Regularly Interspaced Short Palindromic Repeat (CRISPR)/CRISPR-associated protein (Cas) system is one of the genome editing technologies useful in functional genetic research, biotechnological applications, and medical research. Genome editing technology is a technology for precisely targeted modification of endogenous genes in organisms [1]. A specific endonuclease is used to cut DNA strands to achieve the insertion, deletion, and replacement of specific target DNA sequences [2]. Using this, researchers can edit multiple specific sequences efficiently and economically and change or eliminate the molecular functions of target genes. In the recent methodology development history, there have been three major phases, including Zinc Finger Nucleases (ZFNs) [3,4], Transcription Activator-Like Effector Nucleases (TALENs) [5,6], and CRISPR/Cas [1]. Of these, the CRISPR/Cas system is the latest and has developed rapidly owing to its easy operation, high gene editing activity, and the ability to edit multi-targets, thereby becoming a method of choice for genome editing [7].

## 2. The Origin of the CRISPR/Cas System

In 1987, Ishino et al. first discovered an unusual repetitive DNA sequence that formed five copies of tandem repeats at the 3′ end of the alkaline phosphatase (*AP*) isoform converting enzyme gene (*iAP*) in *Escherichia coli* [8]. Subsequently, more researchers found that this multi-repeated palindromic sequence is widespread in the genomes of bacteria and archaea. Using various bioinformatic analyses, Mojica and colleagues successively discovered repeated short sequences with similar structures in dozens of microorganisms and named them short regularly spaced repeats (SRSRs) [9]. This indicated that SRSR might be ubiquitous in the genomes of prokaryotes, including all thermophilic bacteria and archaea, as well as some cyanobacteria and proteobacteria. Later, the SRSR was found to contain 24–40 bp short palindromic repeat sequences organized in clusters and separated by non-repetitive 20–58 bp sequences [9,10]. In 2002, Jansen and colleagues named this sequence ‘Clustered Regularly Interspaced Short Palindromic Repeats (CRISPR)’ [11]. It has since been confirmed that most prokaryotes have two or more CRISPR leader sequences, and the front ends of these sequences share a conserved sequence (300–500 bp) across species [11]. Further studies revealed that a class of CRISPR-associated protein (Cas) genes, which encode endonucleases, existed near the CRISPR sequence, and the bacterial Cas proteins cleaved the exogenous DNA during phage infestation [12]. It turns out that this is an immune-like defense system of prokaryotes to combat the invasion of exogenous DNA, such as that from viruses [13].

## 3. The Basic Structure and Function of the CRISPR/Cas System

**Figure 1:**
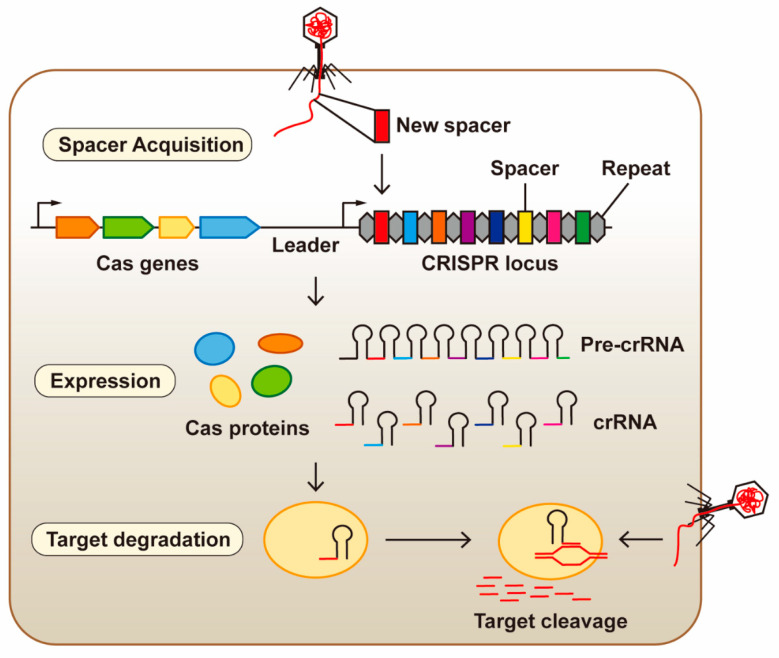
Overview of the CRISPR–Cas immune system. Spacer acquisition: the spacer sequence from the virus is sampled and then integrated into the CRISPR locus. Expression: Pre-crRNA is transcribed from the leader region and processed into smaller crRNAs by Cas proteins. Target degradation: the crRNA and Cas endonuclease complex identifies invading nucleic acid (viral or plasmid) sequences and initiates a cleavage event.

As illustrated in Figure 1, the CRISPR/Cas system is composed of CRISPR sequences (including leader sequences, spacer sequences, and palindromic repeat sequences), Cas endonucleases, and pre-CRISPR RNA (pre-crRNA) [14,15,16,17]. The leader sequence is a conserved sequence associated with transcription located upstream of the CRISPR sequence. The spacer sequence is derived from viral or plasmid nucleic acid and is generally used as a recognition element to search for the matching sequence in the invaded DNA in order to destroy it. The palindrome repeat sequence plays a decisive role in the position and direction of the spacer sequence at the target site [18]. In the downstream region of the target site, there is the protospacer adjacent motif (PAM), a 2–6 bp specific sequence (canonically 5′-NGG-3′, where N represents any base), which serves as the recognition site of the Cas endonuclease [19].

More than 40 Cas proteins have been identified in prokaryotic genomes. They have been shown to function in the formation of CRISPR RNA (crRNA) and the integration and shearing of foreign DNA [20] (Figure 1). Based on the number, type, and characteristics of the Cas proteins, the CRISPR/Cas system can be categorized into two classes, namely Class 1 and Class 2 [19]. The Class 1 CRISPR/Cas system contains multiple Cas proteins and is mainly distributed in bacteria and archaea, while the Class 2 CRISPR/Cas system contains only a single Cas protein and has so far only been found in bacteria. The Class 1 CRISPR/Cas system can be further divided into Type I, Type III, and Type IV, while Class 2 includes Type II, Type V, and Type VI. Compared with Class 1, Class 2 system is simpler in structure and easier to modify and apply [21]. The widely used CRISPR/Cas9 is a Type II CRISPR system.

## 4. The Development and Application of the CRISPR/Cas System as a Genome Editing Tool

In 2012, Sternberg et al. initially demonstrated that crRNAs and trans-activating CRISPR RNAs (tracrRNAs) can pair to form a bimolecular RNA structure and mediate the cleavage of target DNA sequences via the Cas9 protein in vitro [22]. Subsequently, CRISPR/Cas9 technology was developed and successfully applied to achieve precise multiplex gene targeting in mammalian cells [2,23]. While the widely used CRISPR/Cas9 system belongs to the Type II CRISPR system, the emerging Cas12a (Cpf1) system, which is similar to Cas9, belongs to the Type V CRISPR system [19]. Both Type II and Type V are the most intensively explored and widely used systems at present.

The most commonly used Cas9 variant for editing genomes of plants, animals, and microalgae is the Cas9 nuclease of *Streptococcus pyogenes*, which is 1368 amino acid residues long [1]. The CRISPR/Cas9 system mainly consists of two components: the single guide RNA (sgRNA) and the Cas9 protein [24]. The function of sgRNA is to recognize PAM in the target sequence and guide the double-stranded DNA cleavage upstream of the PAM. The Cas9 system performs a blunt-end cut, and the break is usually repaired via non-homologous end joining (NHEJ), resulting in random deletions or insertions of several bases, thus disrupting the correct expression of the target gene [1]. However, even though the use of the Cas9 system has been an enormous success, it is not free of off-target errors. To suppress the off-target activity, researchers have developed a series of Cas9 mutants (e.g., Cas9-HF1, eCas9, HypaCas9, nCas9, and fCas9) with modifications in the spatial structure or active site, which result in a significant reduction in off-target rate [25,26,27,28,29]. Cpf1 is an RNA-dependent endonuclease with similar functions but different characteristics than Cas9. Firstly, Cas9 employs two RNA molecules (crRNA and tracrRNA), whereas Cpf1 only has crRNA, as the guide to search for the target. Secondly, Cpf1 recognizes the longer thymine-rich PAM sequence ‘TTTN’, whereas Cas9 identifies the guanine-rich PAM sequence ‘NGG’. Thirdly, Cpf1 protein cleavage produces a sticky end (5′ protruding end) instead of a flat end produced by Cas9 cleavage [30,31]. Normally, Cas9 does not induce mutations twice at the same site, but Cpf1 can cut the target site again at the mutated site, which is more conducive to homologous recombination. Thus, researchers have shown that the CRISPR/Cpf1 system can achieve efficient target recombination and knock-in [32].

**Figure 2:**
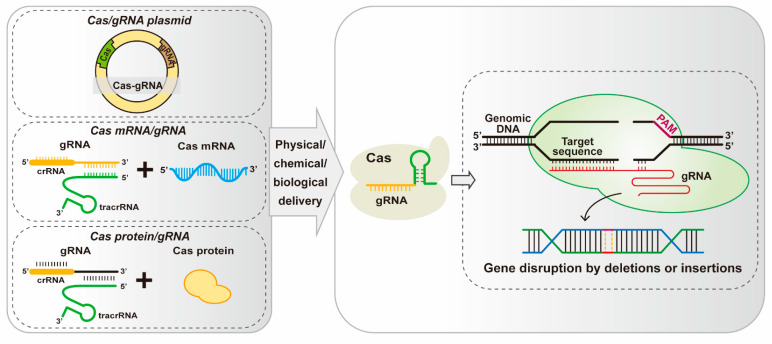
Workflow of CRISPR/Cas-based genome editing.

In addition, the editing efficiency of the CRISPR/Cas system depends largely on the expression of intracellular Cas and guide RNA (gRNA). Several approaches, including plasmid transfection, transfection of in vitro transcribed Cas mRNA and gRNA, and transduction of Cas protein and gRNA complexes (also known as CRISPR-Cas ribonucleoprotein complex [RNP] delivery) [33,34], are available to introduce the CRISPR/Cas system into the target organism (Figure 2). One method works better in one type of organism than in another, and it takes trial and error to find out the most suitable method for a particular species. Plasmid transfection is currently the most widely used; however, it may result in the uncontrolled insertion of foreign genes into the genome [35]. Although the delivery of Cas mRNA and gRNA avoids the occurrence of fragment insertion caused by exogenous vectors, RNA degrades easily, making this system unstable [36]. In contrast, the RNP delivery method can achieve stable and rapid gene editing because it does not require intracellular transcription and translation [37]. Similarly, several methods exist for delivering these constructs into target organisms, including physical (gene-coated particle bombardment, electroporation, microinjection, etc.), chemical (liposome, polyethylene glycol, etc.), and bacterial-mediated delivery methods [35,38,39,40,41] (Figure 2). Which method works the best depends on the characteristics of the target organism. For instance, for the bombardment method to work, the species needs to be able to grow on a solid medium. For microinjection, the target needs to be large enough (e.g., embryos).

The CRISPR/Cas system can also be employed for CRISPR interference (CRISPRi) and CRISPR activation (CRISPRa) by using the DNase dead Cas (dCas) variants and further regulating the gene expression [42]. It has been found that the dCas protein will only have DNA binding ability but no nuclease activity after the two domains of Cas9 (HNH and RucV) are inactivated [43]. Furthermore, the fusion of dCas9 proteins with various transcriptional repressors (e.g., KRAB) or transcriptional activators (e.g., VP64) can repurpose the system for downregulating or upregulating target genes [43,44]. In addition, compared to traditional genome editing methods, the repression effect on gene expression by CRISPRi is reversible, which even allows the simultaneous expression regulation of multiple target genes. Compared with RNA interference (RNAi), which targets mature RNAs in the cytoplasm, CRISPRi prevents the initiation of transcription in the nucleus, achieving significant knock-down effects, which offers broad application prospects in the functional research of genes in various organisms [45].

The utility of the CRISPR/Cas genome editing systems has grown explosively in all fields of biology. The technology has also been a major driving force of innovation in applied and technological areas, including medicine, agriculture, and aquaculture. In medicine, CRISPR/Cas9 technology shows great potential for the construction of animal models and cell lines, treatment of diseases (e.g., cancers, viral infections, and genetic diseases), drug target screening and targeted therapy, although technological challenges associated with off-target editing still need to be solved before clinical applications can become a reality [46,47,48,49]. In agriculture and aquaculture, CRISPR/Cas has been used to improve the yield and quality of crops and cultivated organisms, the resistance against diseases (bacterial and fungal) and pollutants (e.g., herbicides, pesticides). CRISPR/Cas has manifested as a powerful tool in crop and animal breeding and domestication [50]. Similarly, CRISPR/Cas technology has revolutionized the research fields of marine biology and biological oceanography. This review is aimed to assess the past achievements and current challenges of CRISPR/Cas-related research in the global ocean and shed light on a future prospect of the utility of CRISPR/Cas in advancing our understanding of how the marine organisms perform in interaction with each other and with the rapidly changing climate and environment.

## 5. Huge Inventory of CRISPR/Cas in the Ocean

Bacteria and archaea are the most abundant organisms in the ocean, and they all interact at varying magnitudes with viruses and other organisms [51,52]. These microbes have evolved diverse CRISPR/Cas systems to counter invasions of ubiquitous viruses in the environment. Serving as a natural immune mechanism, these CRISPR/Cas systems inform us about how microorganisms interact with ambient viruses and provide a unique window for a deeper understanding of the functioning of marine ecosystems.

Few of the earlier-stage single-strain microbial genome sequencing studies have looked at CRISPR/Cas gene clusters. However, this changed in subsequent years, and the CRISPR/Cas system has recently been documented in the plethora of marine microbial genomes (Table 1). These data indicate that the CRISPR/Cas system exists in many taxonomic groups, functional groups, habitats, and lifestyles (free-living vs. symbiotic) of prokaryotes.

**(1) *Prevalence of CRISPR/Cas in diverse functional and taxonomic groups with taxonomic hotspots.*** As shown in Table 1, all the major phyla of bacteria and archaea deploy the CRISPR/Cas defense system. Some phylogenetically diverse functional groups have a high prevalence of CRISPR/Cas. For instance, a comparative analysis of 91 sulfate-reducing prokaryote (SRP) genomes revealed the existence of CRISPR/Cas systems in as many as 78% of taxa [53]. This frequency is remarkably higher than in other reported prokaryotes. The CRISPR/Cas system is also widespread in some taxonomic groups. *Bacteroidota* shows a remarkable 65% prevalence of CRISPR/Cas system (305 out of 467 complete genomes examined) [54], higher than the average of the bacterial superkingdom (~50%) [19]. Similarly, in *Salinispora* from the phylum *Actinomycetota,* CRISPR arrays were found in all 75 strains surveyed [55]. Meanwhile, most *Salinispora* genomes possess multi-CRISPR-array loci and diverse Cas subtype gene clusters, with some strains harboring up to five different subtypes. In addition, strains isolated from the same location displayed substantial variations in the number of spacers, likely reflecting a diversified strategy to combat different viruses.

Furthermore, the widespread heterogeneity in CRISPR/Cas presence and characteristics occurs within the class γ-proteobacteria. For example, four sympatric strains of the marine *Photobacterium damselae* subsp. within the order *Vibrionales* exhibit pronounced dissimilarities in their CRISPR/Cas systems. These differences range from the coexistence of two distinct CRISPR/Cas systems in certain strains, the presence of only one in others, to the absence of identifiable Cas proteins in some strains based on genomic annotations [56]. Similarly, *Alteromonas macleodii* within the order *Alteromonadales* presents an intriguing contrast regarding CRISPR/Cas systems across different geographical isolates [57]. Specifically, among the sequenced strains of *A. macleodii* isolated from various regions, only the Mediterranean isolate (AltDE) has been found to harbor a CRISPR/Cas system within its genome. Within the same order Alteromonadales, 40% of Shewanella algae strains have been identified with CRISPR/Cas systems and varied greatly in the number of spacer sequences among different strains [58]. In *Nitrosococcus oceani*, the quantity of spacer sequences varies by up to sixfold among diverse strains [59]. These considerable discrepancies might reflect differential susceptibilities to viral attacks among different bacterial strains.

The CRISPR/Cas defense also appears to be important in cyanobacteria, the most important primary producers in the global ocean. In a study examining 126 cyanobacterial genomes, 88.5%, excluding those within the marine subclade (*Synechococcus* and *Prochlorococcus*), were found to harbor CRISPR/Cas systems [60]. Remarkably, within *Geitlerinema* sp PCC 7105 alone, an impressive count of 650 direct repeat-spacer units was identified, distributed among 15 CRISPR loci. In a separate study focusing on multicellular cyanobacteria, the diversity of CRISPR/Cas systems within filamentous cyanobacteria varied significantly across different strains in both the types and numbers of CRISPR/Cas gene clusters [61]. Filamentous marine cyanobacteria such as *Geitlerinema* sp. FC II and *Lyngbya confervoides* Strain BDU141951 contain multiple CRISPR/Cas gene clusters [62,63]. The type III-B CRISPR/Cas systems exhibited a widespread distribution within *Trichodesmium thiebautii* while being absent in *Trichodesmium erythraeum* [64]. This dynamic phenomenon can likely be attributed, at least in part, to the vast diversity of cyanobacterial groups and their global distribution.

**(2) *Absence of CRISPR/Cas in some lineages and alternative defense systems.*** For instance, *Pseudovibrio* from the class *α-proteobacteria* showed a relatively higher reliance on the restriction modification (RM) systems than the CRISPR/Cas system for viral resistance [65]. Among the 18 *Pseudovibrio* strains isolated from sponges, coral, tunicates, flatworms, and seawater, the CRISPR/Cas system was detected only in *Pseudovibrio stylochi* UST20140214-052, a flatworm-associated bacterium. Most *Vibrio* species within the class γ-proteobacteria are equipped with RM systems, but a small number of species possess both the RM and CRISPR system [66], and the frequency of CRISPR defense systems is notably lower than the average of the bacterial superkingdom [54,67]. From the 1935 publicly available *Vibrio* genomes, a screening revealed that CRISPR arrays were present in 278 genomes, with only a 14% prevalence. Within the species *Vibrio parahaemolyticus*, 35% (200 strains) of the 570 strains examined harbor CRISPR/Cas [68]. Despite the relatively low prevalence, CRISPR/Cas systems in *Vibrio* are diverse, with various subtypes [69]. In an investigation of 70 species within the Vibrionaceae, eight distinct CRISPR/Cas types with Cas locus architectural variants were found, highlighting the considerable diversity of Cas protein in this lineage of bacteria [70].

Curiously, the widely distributed marine *Synechococcus* and *Prochlorococcus* lack CRISPR/Cas systems [60]. It has been suggested that this lack could be due to the relatively compact genomes of these organisms, leading them to opt for less genetically burdensome antiviral mechanisms. Some studies on the phage-resistant strains of marine *Synechococcus* and *Prochlorococcus*, however, propose an alternative explanation, i.e., these bacteria might have altered cell surface genes involved in phage attachment [71,72]. Yet, an alternate possibility cannot be excluded, wherein the cost of maintaining a CRISPR/Cas defense system might outweigh the benefits for small-genome species such as marine *Synechococcus* and *Prochlorococcus*.

**(3) *CRISPR/Cas systems in diverse habitats and geographical heterogeneity.*** For instance, the γ-proteobacteria *Zobellella denitrificans* ZD1 and *Marichromatium gracile* YL28, isolated from mangroves, both contain CRISPR/Cas systems, and most of the spacers in the genome of *Z. denitrificans* ZD1 matched double-stranded DNA viruses or unknown phages [73,74]. *Alteromonas* sp. SN2 and *Marinilongibacter aquaticus* YYF0007T from the marine intertidal zone were also found to contain CRISPR/Cas systems [54,75]. In the estuarine ecosystems, the genome of *Vibrio gazogenes* PB1 harbors three CRISPR/Cas operons and four Cas-less CRISPR arrays [76]. *Candidatus Venteria ishoeyi* isolated from the hypoxic waters of the upwelling ecosystem also possesses CRISPR/Cas systems [77].

The deep sea is another rich source of CRISPR/Cas discovery. For instance, the deep-sea bacterium *Salinimonas profundi* strain HHU 13199^T^ exhibits a distinctive I-F-type CRISPR/Cas system [78]. *Varunaivibrio sulfuroxidans* Strain TC8^T^ inhabiting submarine Tor Caldara gas vents of Tyrrhenian Sea [79], *Cycloclassticus* sp. 78-ME [32], an important polyaromatic hydrocarbon-degrading bacterium residing in petroleum deposits, and thermophilic chemolithoautotroph *Desulfurobacterium* sp. strain AV08 and *Thermosulfurimonas* strain F29 from deep-sea hydrothermal environments [80,81] all possess the CRISPR/Cas system.

CRISPR/Cas defense mechanisms have been found in the genome of marine archaea *Nitrososphaerota* and *Nanoarchaeota* inhabiting extreme environments [82,83,84]. Moreover, complex CRISPR/Cas systems have been identified in some archaea within the Asgard superphylum [85], the hyperthermophiles within the phylum *Euryarchaeota* [86,87], and some extremely halophilic archaeons like *Salinigranum rubrum* GX10^T^ and *Halorussus halophius* sp. ZS-3^T^, both belonging to the phylum *Euryarchaeota* [88,89].

Interestingly, comparing the CRISPR/Cas systems in the Arctic and two temperate *Nostoc* species revealed that the Arctic strain possessed a subtype I-B system, which was previously unreported in cyanobacteria [90]. Conversely, in *Nodularia spumigena* isolated from the Baltic Sea (strain UHCC 0039), a similar set of CRISPR/Cas cassette elements is shared with *Nodularia spumigena* CENA596 that was obtained from a shrimp production pond in Brazil [91].

**(4) *CRISPR/Cas systems in bacteria of different lifestyles.*** In addition to the free-living microbes discussed above, the CRISPR/Cas antiviral system has also been reported in bacteria that are associated with other organisms. For instance, 429 spacer sequences within the three CRISPR repeat regions were identified in the genome of the Gram-negative bacterium *Saprospira grandis* str. Lewin, which can capture and prey upon other marine bacteria [92]. In the fish pathogenic bacterium *Streptococcus iniae* SF1, a CRISPR/Cas system containing four Cas genes was identified [93]. A significant percentage (75%, 9 out of 12) of the pathogenic *Moritella viscosa* strains that infect fish mucosa are equipped with CRISPR/Cas systems [94]. *Candidatus Mycoplasma liparidae*, a marine animal gut microbe residing within the Hadal Snailfish from ocean trenches, was found to harbor the CRISPR/Cas system, potentially providing viral protection to its host [95]. CRISPR/Cas system genes have also been identified in strains isolated from sponge-associated microorganisms, such as *Bacillus pumilus* 64-1 and *Thalassoroseus pseudoceratinae* strain JC658^T^ [96,97].

The marine Roseobacter *Monaibacterium* sp. ALG8 associated with brown algae harbors two distinct CRISPR/Cas immune systems [98]. Additionally, CRISPR/Cas systems have been found in epibiotic cyanobacteria such as *Acaryochloris marina* [99] and endosymbiotic cyanobacteria such as *Candidatus Endoriftia persephone* within *Riftia pachyptila* [100]. Remarkably, the discovery of CRISPR spacers matching the phage sequences in *A. marina* constitutes the first report of CRISPR/Cas defense mechanism in a cyanobacterial/cyanophage system. The presence of the CRISPR/Cas system in these two phylogenetically distinct symbiotic cyanobacteria challenges the earlier notion that most symbiotic microorganisms lack CRISPR/Cas systems.

Analyses of marine metagenomics and metatranscriptomics data focusing on the interplay between host CRISPR/Cas systems and phages within marine biofilms unveil an intensified interaction between intracellular viruses and bacteria [101,102]. Considering the dense microbial communities within biofilms, the elevation in viral immunity could be attributed to the quorum sensing mechanism among these microbial communities. This proposition finds support in earlier investigations, where it was observed that the quorum sensing mechanism in the marine prototype bacterium *Chromobacterium violaceum* CV12472 exerts control over the expression of the CRISPR/Cas system [103]. Consistent with the observation, CRISPR arrays found in the Black Sea are predominantly present within the dominant bacterial phyla [104]. Metagenomic analyses have also unveiled an enrichment of genes encoding CRISPR/Cas systems and defense-related mobile genetic elements in microbial communities of sponges. Notably, microbial communities thriving in HMA (high microbial abundance)-like sponges exhibit higher CRISPR/Cas defense capability than those inhabiting sponges with a lower microbial abundance [105]. Metagenomic analyses have further revealed a significantly elevated occurrence of CRISPR/Cas proteins and restriction endonucleases within sponge tissues as compared to the surrounding environment [106,107,108]. This suggests that the microbial communities within sponges require heightened antiviral activity compared to their external environment. Notably, almost all the symbiotic MAGs in the sponge *Bathydorus* sp. SQW35 have genes encoding Cas proteins and an ammonia-oxidizing Nitrososphaerota MAG B01, dominating the internal sponge environment, exhibits a highly complex CRISPR array [109]. This complex array signifies a favorable evolutionary adaptation to a symbiotic lifestyle and reflects a potent ability to resist phage attacks within the sponge’s ecosystem. Interestingly, CRISPR/Cas systems have also been reported from samples of feces in wild marine animals and the deep-sea hagfish gut [110,111].

**(5) *Evolutionary trajectory and driving force are elusive.*** The detection in the wide phylogenetic range of microbes suggests that the CRISPR/Cas antiviral defense mechanism emerged very early in evolution and that these ancient microbes had been exposed to phage infections in their ancient extreme environments. However, its origin and driving force are still unclear. The punctate distribution of CRISPR/Cas systems across different taxa suggests that the defense system either independently arose or was lost due to selection forces. It has been suggested that CRISPR/Cas systems are more widespread in thermophilic prokaryotes than in mesophilic prokaryotes [112]. Moreover, CRISPR/Cas systems have been observed in psychrophilic bacteria, but they appear to be inactive [113]. In a comparative study of three different isolates of *Thalassolituus oleivorans* strains, it was noted that strains from polar marine environments lacked CRISPR Cas systems [114]. Within marine hydrothermal ecosystems, the *Aquificales,* characterized by their exceptionally condensed genomes, show the variable CRISPR/Cas systems and the number of repeats in a cluster [115]. Another study identified the presence of two CRISPR/Cas systems (Type I and Type III) without an RM system in the genome of *Thermosipho affectus* within the phylum *Thermotogota* [116]. These findings suggest that temperature influences the selection of antiviral strategies by bacteria. This trend might be attributed to the fact that mesophilic prokaryotes generally exhibit mutation rates severalfold higher than those of thermophilic prokaryotes [112]. It follows that the wider occurrence of CRISPR/Cas systems among archaea might be attributed to the unique selection pressures associated with archaea, as most of them inhabit high-temperature environments [117]. However, notable exceptions to this “high-temperature” proposition exist, e.g., the Asgard archaea in the Haima cold seep also possess CRISPR/Cas systems [85]. Evidently, it is important to consider the interaction of the genetic background with the environment in an attempt to trace the origin of the CRISPR/Cas system. In addition, some taxa may have developed other antiviral mechanisms (e.g., RM) that reduce their dependence on the CRISPR/Cas system for viral defense.

**Table 1:** Distribution of common types of Cas across major phyla of marine prokaryotes.

Phylum	Cas Type	Species	Prevalence *	References
Pseudomonadota	I-F and I-E	*Moritella viscosa*	Prevalent	[32,57,58,73,74,75,77,78,79,94,98,100,118]
		*Vibrio*	Some species	[56,65,66,69,70,76]
Cyanobacteriota	I-D and III-B	*Geitlerinema* sp. FC II	Prevalent	[63,90,91]
		Marine subclade *Synechococcus* and *Prochlorococcus*	Rare	[60]
Actinomycetota	I-E and I-U	Marine actinomycete *Salinispora*	Prevalent	[55]
Bacteroidota	II-C and VI-B1	*Marinilongibacter aquaticus*	Prevalent	[54]
Thermotogota	III-A and III-B	*Thermosipho* spp.	Limited study	[116]
Aquificota	unclassified	*Aquificaceae*	Prevalent	[115]
Thermodesulfobacteriota	III-B and I-B	*Desulfobacterium*	Prevalent	[53,81]
Bacillota	III-B	*Bacillus pumilus* 64-1	Limited study	[96]
Planctomycetota	unclassified	*Thalassoroseus pseudoceratinae*	Limited study	[97]
Mycoplasmatota	II	Candidatus *Mycoplasma liparidae*	Limited study	[95]
Nitrososphaerota	unclassified	Candidatus *Nitrosopumilus koreensis* AR1	Limited study	[82]
Nanoarchaeota	I-B	*Nanoarchaeum equitans*	Limited study	[83]
Euryarchaeota	I-A and III-B	*Pyrococcus furiosus*	Prevalent	[86,87,88,89]
Asgard group	I-A and III-B	Candidatus *Thorarchaeota archaeon*	Metagenome-assembled genomes	[85]

* ‘Prevalent’ signifies a frequency of occurrence exceeding the average observed within the bacterial superkingdom (~50%); ‘Some species’ indicates a frequency lower than the average observed within the bacterial superkingdom; and ‘Rare’ signifies existence only in some species. In the case of phyla with limited research coverage, they are designated as ‘Limited study’.

**(6) *Emerging novel CRISPR/Cas systems***. As research delves deeply into CRISPR/Cas systems in marine microbes, more CRISPR/Cas systems continue to be found. The discovery of novel Cas protein structures and functions can advance the development of more efficient gene editing systems and enhance our understanding of how CRISPR/Cas systems are regulated. Particularly noteworthy is that *Emcibacter congregatus* ZYL^T^, isolated from sediment samples, possesses a complete II-C type CRISPR/Cas system, with its predicted Cas9 protein being markedly smaller than the majority of existing genome editing tools [118] and a diverse range of predicted Cas9 proteins have been identified within the oral microbiomes of marine mammals, such as dolphins, including two of the longest predicted Cas9 proteins reported to date [119]. Moreover, a fusion between Cas1 and reverse transcriptase has been reported in the marine bacterium *Marinomonas mediterranea*, enabling a host-mediated reverse information flow from RNA to DNA [120]. A similar phenomenon of Cas protein fusion with reverse transcriptase domains is also prevalent in cyanobacteria [61].

Marine microbes also provide resources for further understanding this ancient antiviral system and insight into the arms race between viruses and hosts. CRISPR spacer tends to target crucial viral genes involved in replication, nucleic acid binding, and viral structural proteins essential for infection [121]. This targeting specificity may imply a pattern of co-evolution. Specifically, phages subjected to CRISPR/Cas system surveillance can evade detection by undergoing simple mutations and deletions within the protospacer adjacent motif and spacer region. This escape mechanism serves as a means of countering host CRISPR/Cas system resistance. The response mounted by host cells does not rely on a singular spacer sequence for viral defense; instead, multiple CRISPR spacers often target the same virus [122]. Moreover, this evasion strategy can be countered in certain marine bacteria by introducing other types of CRISPR/Cas systems, effectively ‘chasing’ the escaped viruses. Just as observed in *Marinomonas mediterranea*, viruses that have managed to escape the defense of I-F type CRISPR/Cas systems due to genetic mutations in the PAM are subsequently captured by III-B type CRISPR/Cas systems that utilize spacers from the I-F type system [123]. During interception, phages may adopt innovative strategies to elude the host’s CRISPR system, such as encoding RNase T with potential functionalities to enable the mark-up or digestion of the crRNA [99].

Viruses employ not only passive gene mutations for evasion but also various active escape mechanisms. In phage CL 131, a putative type V-U2 CRISPR/Cas system is encoded [122], which carries spacers targeting the host cyanobacterial genome, and certain phage CRISPR/Cas systems possess the capability to silence host transcription factors and translation genes [124]. Moreover, apart from evading the CRISPR-based interception by host cells, viruses also exhibit the ability to generate spacers targeting other phages. This strategy potentially bestows the host with immunity against other phages, consequently affording the host a competitive advantage [125]. This interplay of capture and evasion contributes to the co-evolution of phages and hosts [126]. In marine *Salinispora*, instances of CRISPR/Cas systems targeting their own genomic sequences have been discovered, which might be involved in the self-regulation of metabolism, but experimental evidence has yet to be attained [55]. The diverse and complex marine environment offers a wealth of paradigms for investigating the ancient antiviral system and developing biotechnological tools.

## 6. Applications on Marine Prokaryotic Microbes

**(1) *Microbial genome editing using CRISPR/Cas.*** CRISPR/Cas technology presents a powerful tool capable of concurrently editing all chromosome copies in marine microorganisms. Therefore, successful genome editing using the CRISPR/Cas system has been achieved in various aquatic microbes (Table 2). Comparatively, the applications of CRISPR/Cas9 to freshwater microbial research appear to be more advanced than to marine microbes [127,128]. Marine microorganisms are more recalcitrant to genetic engineering than freshwater microorganisms, as is evident in knocking out biosynthetic gene clusters in actinomycetes [129]. This resistance may be attributed to the co-evolution with more diverse viruses in the marine environment. Despite these limitations, a dual-function chromogenic screening-based CRISPR/Cas9 genome editing system has been established and used to obtain the precise knockout of the carotenoid gene cluster and the abyssomicin gene cluster in marine *Verrucosispora* sp. MS100137 within the phylum *Actinomycetota* [130]. Furthermore, a single plasmid encompassing CRISPR/Cas9 and RecE/RecT recombinase was employed for genome editing in *Shewanella algae* from the phylum *Pseudomonadota*, which is a widespread source of antibiotic resistance in marine environments [131]. This approach effectively reversed carbapenem resistance in *S. algae*.

**(2) *Retailoring CRISPR/Cas to CRISPRi for gene knockdown.*** To address the challenges posed by Cas9-induced cell toxicity and subsequent editing failures, numerous CRISPR/dCas systems were developed. These systems involve deactivated Cas9 proteins that lack DNA cleavage activity positioned within the genome to enable the precise regulation of gene expression. Functional CRISPRi was used to knockdown *vioA* and *macB* genes in *Pseudoalteromonas luteoviolacea*, genes involved in violacein biosynthesis and the formation of the MACs complex that triggers tubeworm metamorphosis, respectively [132]. The CRISPRi technique facilitates studies of the interactions between marine hosts and microorganisms and the transformation of twelve marine strains across two Proteobacteria classes, four orders and ten genera [132]. The CRISPR/dCas9 interference technique has also been applied in *Vibrio fluvialis*, revealing the crucial role of *torA*, encoding one of the catalytic subunits of trimethylamine N-oxide (TMAO) reductase, in conferring the tolerance of high hydrostatic pressure [133]. The CRISPRi technique has also been applied in *Vibrio natriegens* for functional gene screening, as the CRISPR/Cas9 system alone was inadequate for efficient mutant generation through NHEJ-based gene knockout [134]. The work led to the identification of 587 genes as ‘core genes’ required for rapid growth in rich media. Furthermore, *V. natriegens* developed a novel NT-CRISPR approach by expressing anti-Cas9 proteins to overcome Cas9-induced cell toxicity, generating up to 100% editing (deletion) efficiency [135]. Recently, advancement was reported in the utilization of CRISPR/dCas9 for precise and efficient single-nucleotide resolution genome editing in the aromatic compound-metabolizing bacterium *Roseovarius nubinhibens* [136]. This was achieved without inducing double-strand breaks or the provision of donor DNA. Leveraging the editing system, critical genes within the β-ketoadipate pathway unequivocally establish the indispensability of *pobA*, *pcaH*, and *pcaG* in the β-ketoadipate pathway for the degradation of aromatic compounds [136]. Moreover, this study revealed that the absence of *pcaQ* in the genome hinders the consumption of aromatic compounds.

Besides the phyla *Actinomycetota* and *Pseudomonadota*, the application of CRISPRi technology has been explored in the marine cyanobacterium *Synechococcus* sp. strain PCC 7002 [137]. This endeavor achieved conditional and titratable repression of the heterologous expression of fluorescent proteins, which were harnessed to modulate two crucial proteins involved in the photosynthetic processes in *Synechococcus* sp. Similarly, the CRISPR/Cas9 genome editing system has also been established in archaea as a significant component of prokaryotic organisms. Within *Methanococcus maripaludis*, utilizing the CRISPR/Cas9 genome editing system yielded highly efficient (75–100%) and precise genome modifications [138]. This technique enabled in situ mutations within the genome, including successfully deleting *MMP0431* and *MMP1381* genes encoding putative members of the ribonucleases β-CASP family. The dispensability of these genes within *M. maripaludis* was established. As CRISPR gene editing systems require the simultaneous expression of both Cas9 protein and sgRNA, the endogenous CRISPR defense systems of microbes should be explored to achieve efficient gene editing.

**Table 2:** The application of CRISPR/Cas in marine prokaryotic microbes.

Taxonomy	Species	CRISPR Systems	Application	References
Euryarchaeota	*Methanococcus maripaludis*	CRISPR/Cas9	Deleted multiple genes across different loci; in situ genome modifications	[138]
Actinomycetota	*Verrucosispora* sp. MS100137	CRISPR/Cas9	Deleted orange-pigmented carotenoid gene cluster and abyssomicin gene cluster	[130]
Pseudomonadota	*Shewanella algae*	CRISPR/Cas9	Deleted potential carbapenem resistance genes	[131]
	*Vibrio natriegens*	NT-CRISPR (CRISPR/Cas9)	Deletions, integrations and single-base modifications	[135]
	*Vibrio natriegens*	CRISPR/dCas9	Genome-wide CRISPR interference	[134]
	*Vibrio fluvialis*	CRISPR/dCas9	CRISPR interference *torA* gene	[133]
	*Pseudoalteromonas luteoviolacea*	CRISPR/dCas9	CRISPR interference the *vioA* gene and *macB* gene	[132]
	*Roseovarius nubinhibens*	CRISPR/dCas9	*PobA*, *pcaH*, and *pcaG* in the β-ketoadipate pathway, and a transcription activator pcaQ	[136]
Cyanobacteriota	*Synechococcus* sp. strain PCC 7002	CRISPR/dCas9	Achieved conditional and titratable repression of gene	[137]

**(3) *Use of CRISPR as a marker to track microbe-virus interactions in the ocean.*** Strikingly, comparative analysis of CRISPR sequences in prokaryotic genomes and viral genomes enables the identification of host–virus pairs, uncovering new infection relationships, and analyzing virus–host interactions [59,90,104,109,139,140,141,142,143]. For instance, *thy A* gene, a fragment of the cold-active *Colwelliaphage* 9A, was discovered in the CRISPR sequences of *Syntrophus aciditrophicus*, suggesting a history of viral infection in the organism [113]. Analysis of CRISPR spacer sequences from bacteria isolated in different regions allowed researchers to determine the distribution range of phages capable of infecting these species [57,144]. Similarly, an uncultured Mediterranean phage segment matched with five spacers from *Klebsiella pneumoniae* and two spacers from *Pseudomonas aeruginosa*, implying a broad host range for the phage [145]. CRISPR sequences can also serve as discriminative genes for evolutionary branches, showcasing their potential as tools for bacteria subtyping [146,147,148,149].

CRISPR cassettes can retain memories of local viral populations in particular marine locations [150]. Therefore, these CRISPR systems can serve as specific markers to map viral distribution in the environment and discover novel viruses. Indeed, CRISPR/Cas genes have informed the presence of viruses in modern stromatolites, providing insights into ancient marine environments [151]. New archaeal viruses have been discovered in the marine environment and have been successfully matched with different lineages of archaeal hosts [152]. Surveys of CRISPR/Cas spacers in the North Sea over two consecutive years revealed that certain phages constitute stable components of the North Sea microbial community [153]. Moreover, CRISPR sequences within prokaryotes can serve as tools to unravel the complexity of microbial communities within metagenomic data [154]. They can be employed as genetic markers with substantial capabilities for analyzing the genetic structure of uncultured bacterial populations [155].

## 7. CRISPR/Cas in Marine Algal Research

The rapid advancement of the CRISPR/Cas9 gene editing tool has set off an upsurge of application in eukaryotic algae. Jiang et al. made the first attempt in terms of CRISPR/Cas-based gene editing in the freshwater alga *Chlamydomonas reinhardtii* and achieved a transient expression of Cas9 and sgRNA genes [156]. However, the expression of Cas9 caused cell toxicity and resulted in the low mutagenicity in *C. reinhardtii*. Later, the development of methods led to the improvement of the rate of knockout (~1 knockout/10^7^ cells) [157] and significant phenotypic changes in *C. reinhardtii* mutants [158,159]. At about the same time, CRISPR/Cas9-based gene editing succeeded in the diatom *Phaeodactylum tricornutum* and *Thalassiosira pseudonana* [160,161]. Since then, the CRISPR/Cas9 system has been widely applied for various functional genetic research related to nutrient regulation, photosynthesis process, and algal lipid metabolism in phytoplankton (Table 3).

**(1) *Algal nutrient regulation.*** Recent studies of nutrient regulation in marine phytoplankton (e.g., cellular metabolic regulation of N, P, Si, and Fe) have enormously benefited from the successful application of CRISPR/Cas9 gene editing. One of the nutrients that have been studied using CRISPR/Cas9 gene editing was nitrogen. Using the CRISPR/Cas9 system, Hopes et al. reported the first successful editing of the urease gene in diatom *T. pseudonana*. A decrease in growth rate was observed after the knockout of the urease gene, indicating the impairment of the gene function and potential utilization of alternative N sources in *T. pseudonana* [161]. The knockout of the nitrate reductase gene (*NR*) was performed in *T. pseudonana* using CRISPR/Cas, and the mutants showed suppressed growth on nitrate [162]. Recently, CRISPR/Cas9 technology was successfully applied to the nutrient regulation study in the centric diatom *Chaetoceros muelleri* [163]. The knockout of *NR* and urease genes generated single- and double-knockout lines in *C. muelleri*, which led to an auxotrophic phenotype under different N nutrient conditions, providing a useful tool for future studies on *C. muelleri* [163]. In green alga, CRISPR/Cas9 technology was also used to construct auxotrophic strains of *C. reinhardtii* [164]. The authors applied the pre-N-starvation to improve the gene editing efficiency (from 10% to 66%), and the loss-of-function mutants of the spermidine synthase gene (*SPD1*) could grow stably under a very low spermidine level (0.75 mg/L), demonstrating the CRISPR/Cas gene editing as a useful tool of auxotrophic marker selection [164].

P-nutrient regulation strategies in marine phytoplankton have also been characterized using CRISPR/Cas9 gene editing. By knocking out the Myb-like transcription factor phosphate starvation response regulator (*PHR*) in diatom *P. tricornutum*, Sharma et al. revealed the important role of *PHR* in algal P acquisition, P scavenging, and phospholipid remodeling during the adaptation to P-limitation [165]. Furthermore, *SPX* in *P. tricornutum* was also modified using CRISPR/Cas9 knockout to reveal its role as a potential upstream negative regulator of P-nutrient homeostasis regulation [166]. The elevated expression of *AP*, phosphate transporters (*PT*), and phospholipid hydrolases after *SPX* knockout indicates that the functional loss of *SPX* promotes P acquisition and phospholipid metabolism [166]. Furthermore, comparing the transcriptomes of the mutants with that of the wild type indicates that *SPX* regulates P uptake in *P. tricornutum* via the *PHR* intermediate. These findings suggest that SPX-PHR is a coupled regulatory cascade of *AP*s and *PT*s and part of a crucial P homeostasis regulation mechanism operating in the diatom living in fluctuating P environments [166]. Subsequently, functional studies were conducted on *AP* using CRISPR/Cas9-based gene mutagenesis [167,168,169]. Phytoplankton can scavenge dissolved organophosphate (DOP) with the aid of *AP* [170,171]. *AP* occurs in multiple isoforms (e.g., *PhoA*, and *PhoD*), and CRISPR/Cas9 was employed to create *PhoA* and *PhoD* mutant lines of *P. tricornutum* [167]. Based on the physiological and molecular analyses of mutant strains under DIP deficiency, the differential expression and DOP substrate specificities of *PhoA* and *PhoD* type *AP*s in *P. tricornutum* were observed, shedding light on the functional differentiation and complementation of *AP* in marine diatoms [167]. Meanwhile, researchers also investigated the function of *PhoA* and *PhoD* in *P. tricornutum* besides P scavenging under P-replete environments using CRISPR/Cas9-based mutants [168,169]. These studies suggest that *PhoA* and *PhoD* in diatoms play roles in constraining pigment biosynthesis, photosynthesis, cell division, and lipid accumulation and maintaining nutrient homeostasis when DOP scavenging is not required [168,169]. Furthermore, You et al. investigated the function of one trypsin gene in *P. tricornutum* (*PtTryp2*) using CRISPR/Cas9-mediated knockout, as well as trypsin overexpression, and monitoring the N acquisition and P uptake after the loss or amplification of *PtTryp2* function [172]. This study indicates that *PtTryp2* is a coordinate regulator of cellular stoichiometric homeostasis in the diatom.

The cell wall protein silacidin is responsible for silica precipitation in the cell wall, and the biallelic replacement of the silacidin gene in *T. pseudonana* using CRISPR/Cas-mediated knockout successfully links the genotype and phenotype and suggests the role of the silacidin gene in regulating the cell size of centric diatoms [162]. In addition, as the first identified silica deposition vesicles (*SDV*) transmembrane protein in diatom, the function of silicanin-1 (*Sin1*) was also unraveled using a CRISPR/Cas9-based approach [173]. Reduced biosilica content and morphological aberrations were observed in the *Sin1*-mutated *T. pseudonana* cells, providing evidence that Sin1 could highly influence the strength and stiffness of cell walls [173].

Iron uptake is crucial for phytoplankton growth and the global biogeochemical cycles of carbon and has thus been extensively studied. The application of CRISPR/Cas9 technology in phytoplankton allowed researchers to reveal the Fe acquisition system at the molecular level [174]. Based on the knockout cell lines of three genes involved in ferrisiderophore acquisition (*FBP1*, *FRE1*, and *FRE2*), a soluble Fe uptake model in *P. tricornutum* was developed [174]. In *T. pseudonana*, CRISPR/Cas9-based knockout was successfully applied to characterize the function of flavodoxin, a functional homologue of Fe-containing Fd [175]. This study indicates that clade II flavodoxin acts on the acclimation to Fe-limitation while the hypersensitivity of clade I flavodoxin mutant lines to H_2_O_2_ certifies the role of clade I flavodoxin in the oxidative stress response other than Fe-starvation adaption in the diatom [175].

**(2) *Algal photosystem.*** CRISPR/Cas9 gene editing was initially applied to determine the function of photosynthesis-related genes in the freshwater alga *C. reinhardtii* [158,176]. The chloroplast signal recognition particle (CpSRP) pathway is crucial for targeting the light-harvesting complex proteins (*LHC*) to the thylakoid membranes [177]. To characterize the CpSRP pathway, the CpSRP receptor (*CpFTSY*) and zeaxanthin epoxidase (*ZEP*) gene in *C. reinhardtii* were disrupted using a DNA-free CRISPR/Cas9 method [158]. Dual-gene (*CpFTSY* and *ZEP*) knockout led to greater photosynthetic activity and zeaxanthin production in *C. reinhardtii* [158], implying the wide prospect of CRISPR/Cas9-induced mutation in environmentally friendly biotechnology [176]. The functions of genes involved in the CpSRP pathway were also investigated in diatoms using CRISPR/Cas9 technology [178,179]. The functional loss of one member of the CpSRP pathway, CpSRP 54 kDa (*CpSRP54*), in *P. tricornutum* led to the decreased accumulation of chloroplast-encoded photosynthetic complex subunits, indicating that *CpSRP54* acts in the co-translational part of the CpSRP pathway in *P. tricornutum* [178]. However, the *LHC* and pigment contents did not decrease in *CpSRP54* mutant lines as plants and green algae do, emphasizing the different pathways for the integration of thylakoid membrane proteins between plants, green algae, and diatoms [178]. Correspondingly, the phenotype of *CpFTSY* mutants created by using the CRISPR/Cas9 system also indicates that *CpSRP54* and *CpFTSY* of the CpSRP pathway have not yet evolved post-translational functions in diatoms [179]. In addition, Sharma et al. attempted to simultaneously introduce indels in multiple *Lhcf* genes in *P. tricornutum*, and the visible color changes of mutant lines demonstrated the successful modification [180]. Meanwhile, the CRISPR/Cas9-based gene knockout of *Lhcx2* was constructed to learn the energy-dependent fluorescence quenching (qE) photoprotection in this diatom [181]. This study indicates that the upregulated *Lhcx2* contributes to the active qE in *P. tricornutum* under Fe-limitation, but other Fe-starvation symptoms were not influenced by *Lhcx2* and qE [181]. Recently, the function of *Lhcf15* in *P. tricornutum* was investigated using CRISPR/Cas9 gene knockout [182]. The depressed growth of loss-of-function mutants under red light indicated that the *Lhcf15* in diatoms is employed to adapt to longer wavelength light environments [182].

CRISPR/Cas9 genome editing has also been used to study photosynthetic pigment metabolism. CRISPR/Cas9 was recently employed to knockout the candidate genes of Chl *c* synthase (*CHLC*) in *P. tricornutum*, resulting in the identification of a previously unsuspected gene as the *CHLC* responsible for the biosynthesis of Chl *c* [183]. Yang et al. investigated the role of cryptochrome (*CryP*), a blue light-sensitive protein, on the fucoxanthin biosynthesis in *P. tricornutum* via CRISPR/Cas9 [184]. This study shows that *CryP* is involved in the regulation of *LHC* expression and carotenoid biosynthesis in the diatom, and *CryP* mutants can be a suitable candidate for fucoxanthin production [184]. The knockout of the antagonistic enzymes violaxanthin de-epoxidase gene (*VDL2*) and zeaxanthin epoxidase gene (*ZEP1*) in *P. tricornutum*, which are essential for the fucoxanthin pathway in diatoms, assisted researchers to complete the fucoxanthin biosynthesis pathway and reveal the diadinoxanthin metabolism as the regulation center between the photoprotective xanthophyll cycle and fucoxanthin formation [185]. In addition, the knockout of the β-carotene hydroxylase gene in *Dunaliella salina* (*Dschyb*) using the CRISPR/Cas9 system led to a 2.2-fold increase in the production of β-carotene [186]. In red algae, the CRISPR/Cas9 gene editing method was successfully used to investigate the function of chlorophyll synthase in *Porphyridium* (*Chs1*), and the mutants showed increased phycoerythrin contents [187].

To investigate the C4 pathway in marine phytoplankton, Huang et al. performed CRISPR/Cas9-based gene editing on the pyruvate orthophosphate dikinase (*PPDK*), a key enzyme generating the primary acceptor for bicarbonate fixation in the C4 pathway, in *P. tricornutum* [188]. The *PPDK* mutant exhibited depressed growth and photosystem II relative electron transport rate (rETR_PSII_) [188]. Combined with gene editing and chlorophyll fluorescence analyses, this study indicated the essential function of *PPDK* in the pH homeostasis maintenance of the diatom [188].

**(3) *Algal lipid production.*** In the face of drastic climate change and energy depletion, algae hold a high potential as a green, renewable, economical, and non-toxic alternative energy sources [189]. Microalgae grow rapidly, and some species have oil contents as high as 75% of dry weight, making them promising species for renewable biodiesel [190,191,192]. To improve the oil yield, researchers have applied the CRISPR/Cas9 gene editing technique to enhance the algal lipid contents [193].

The knockout of genes involved in carbon metabolism, fatty acid (FA) or lipid metabolism by using CRISPR/Cas9 technology has been reported in green algae and diatoms since 2017. The first attempt was the knockdown of the phosphoenolpyruvate carboxylase (*PEPC*) gene in the freshwater alga *C. reinhardtii* through the use of CRISPRi [194]. The functional loss of *PEPC* led to a 74% increase in lipid content and a 94% enhancement of lipid productivity in *C. reinhardtii* [194]. The CRISPR/Cas9-based knockout of the phospholipase A2 gene induced up to 64% enhancement on lipid productivity and increased TAG accumulation in *C. reinhardtii* [195]. Moreover, *C. reinhardtii* mutant strains of an esterase lipase thioesterase (*ELT*) gene involved in FA degradation were generated using CRISPR/Cas9 [196]. The *ELT* mutation led to a 6% increase in lipid proportion in dry weight and a 27% increase in C18:1 proportion in FA, indicating the potential of disrupting lipid catabolism via gene editing to construct high-yield performance microalgal strains [196]. Initial CRISPR/Cas9 editing attempts in the commercially important freshwater alga *Chlorella vulgaris* were successful in knocking out omega-3 fatty acid desaturase (*Fad3*), which caused a 46% enhancement (*w*/*w*) in lipid accumulation [197]. Similar work has been reported in diatoms. Plastidial ACP Δ9-desaturase (*PAD*) is a key enzyme in FA modification and the knockout of *PAD* via the CRISPR/Cas9 system changed the synthesis of long-chain poly-unsaturated fatty acids (LC-PUFA), especially eicosapentaenoic acid (EPA), in *P. tricornutum*, indicating a key role of *PAD* in the regulation of EPA levels [198]. Meanwhile, CRISPR/Cas9 was employed to investigate the function of Δ6 fatty acid elongase in the EPA-rich marine alga *Nannochloropsis oceanica* (*NoΔ6-FAE*) [199]. The increase in C18:3Δ^6,9,12^ but decrease in C20:3Δ^8,11,14^, C20:4Δ^5,8,11,14^, and EPA in *NoΔ6-FAE* mutant lines indicated the involvement of *NoΔ6-FAE* in the EPA biosynthesis via the ω6 pathway in *N. oceanica* and further demonstrated the potential of CRISPR/Cas9 technology to modify lipid composition [199]. In addition, Hao et al. generated mutant lines of a long-chain acyl-CoA synthetases (*LACS*) gene in *P. tricornutum* [200]. The *LACS* mutation influenced algal growth and TAG content and altered FA profiles in galactoglycerolipids and phosphatidylcholine (PC), revealing the functions of *LACS* isozymes in the lipid metabolic process of oleaginous diatom [200]. The use of CRISPR/Cas gene editing unveiled the regulatory role of MGDG synthase (*MGD*) in the synthesis of monogalactosyl diacylglycerol (MGDG), the most abundant polar lipid in the thylakoid membrane, in *P. tricornutum* as the knockout of *MGD* resulted in decreases in MGDG and DGDG (synthesized from MGDG) contents in *P. tricornutum* [201]. The lipid accumulation could form lipid droplets (LDs) in microalgae, and the size and structure of LD were related to the LD protein (*LDP*) [202]. The role of one of the most abundant *LDP*s, stramenopile-type LDP (*StLDP*), was investigated in diatom using CRISPR/Cas9-mediated genome editing [203,204]. The *StLDP* mutants showed an expansion in LD size and a decrease in LD number per cell under N-depleted conditions, indicating the role of *StLDP* as an LD scaffold to regulate LD size and lipid homeostasis in *P. tricornutum* [203,204].

Genes for other functions have also been edited via CRISPR/Cas9 to improve lipid yields in microalgae. The knockout of ADP-glucose pyrophosphorylase (*AGP*), the key regulatory enzyme of starch synthesis, in the marine green alga *Tetraselmis* sp. was achieved by using the DNA-free CRISPR/Cas9 method [205]. The mutant lines showed 2.7–3.1-fold increases in total lipid content and a 5-fold increase in monounsaturated fatty acid oleic acid (C18:1) content [205]. In addition, the CRISPR/Cas9 system was applied to the green alga *Parachlorella kessleri*, and the knockout of a plastidic ATP/ADP translocases (*PkAATPL1*) led to a 30% higher lipid production while the duplicated mannanases 1 (*PkDMAN1*) mutation caused a decrease in growth [206]. The disruption of both glutamine synthetase 2 (*GS2*) and *PhoD* genes through the use of CRISPR/Cas9 led to the increased lipid contents and modified lipid composition of *P. tricornutum* [168,207]. Moreover, the knockout of one novel gene (*Pt2015*) using the CRISPR/Cas9 method also unexpectedly achieved a slight rise in lipid contents in *P. tricornutum* [208]. All these outcomes highlight the enormous potential of CRISPR/Cas9 genetic engineering in bioenergy.

**(4) *Other applications of CRISPR/Cas9 in marine algal research.*** In addition to the applications summarized above, CRISPR/Cas9 gene editing technology has been applied in a wide range of phytoplankton and seaweed research. To improve the precision of the CRISPR/Cas9 system in diatoms, Nawaly et al. developed the Cas9 nickase (D10A) and dual sgRNA system and successfully achieved the mutants of a putative θ-type carbonic anhydrase (*CA*) in the centric diatom *T. pseudonana* with short biallelic indels and low off-target effects [209]. Successful green fluorescent protein (*GFP*) knock-in guided by CRISPR/Cas9 in *T. pseudonana* achieved high efficiencies (>50%) of endogenous *GFP* tagging and the precise creation of *GFP* fusion proteins, providing a versatile toolbox for future functional studies [210].

To investigate the diversity of DNA methyltransferase (*DNMT*) gene in marine phytoplankton, Hoguin et al. mutated the *DNMT5a* gene using CRISPR/Cas9 and demonstrated that the functional loss of *DNMT5* was responsible for the global depletion of DNA methylation and the overexpression of young transposable elements (TEs) in the diatom *P. tricornutum* [211]. CRISPR/Cas9 technology was also used to understand the regulation of programmed cell death (PCD) of marine phytoplankton [212]. Metacaspases could regulate PCD in plants [213], and decreased metacaspase activity was observed after the CRISPR/Cas9-based knockout of a type III metacaspase in *P. tricornutum* [212]. In addition, to better understand the signaling mechanism in phytoplankton, the CRISPR/Cas9 system was used to disrupt a single domain voltage-gated channel (*EukCatA*) in *P. tricornutum* [214]. The result showed that *EukCatA* played critical roles in voltage-regulated Ca^2+^ signaling and Ca^2+^-dependent gliding motility and potentially served as an alternative mechanism of 4D-Ca_v_/Na_v_ channels in pennate diatoms [214]. The CRISPR/Cas9 method was employed to investigate the thiamine metabolic process in *P. tricornutum* [215]. The knockout of the HMP-P synthase (*THIC*) gene (*PtTHIC*) and thiamine-related proteins SSSP gene (*PtSSSP*) indicated that the *PtTHIC* was essential for thiamine biosynthesis while *PtSSSP* served in thiamine uptake [215].

In the model green alga *C. reinhardtii*, the acetolactate synthase (*ALS*) mutation through the use of CRISPR/Cas can create sulfometuron methyl (herbicide) resistance [157]. In addition, CRISPR/Cas9 gene silencing technology was also successfully applied in other green algae, including *Ulva prolifera*, *Picochlorum celery*, and *Volvox carteri* [216,217,218]. Meanwhile, the adenine phosphoribosyl transferase gene (*APT*) in brown algae *Ectocarpus siliculosus* (*Ectocarpus* 7) and *Saccharina japonica* was successfully targeted through the use of CRISPR/Cas9 genome editing, demonstrating the potential of CRISPR/Cas in functional genetic research on brown algae [219,220]. CRISPR/LbCas12a-based gene editing was also achieved in the red macroalga *Gracilariopsis lemaneiformis* for *CA* and γ-subunit of phycoerythrin (*γpe*) [221].

Haptophytes are another group of phytoplankton that are abundant in marine environments and include the calcifying lineage coccolithophores. For the most widespread and abundant coccolithophore *Emiliania huxleyi*, a particle bombardment method was conducted using the constructed vector PnpUC originally derived from pUC18 [222]. Then, *Agrobacterium*-mediated stable DNA transfer into the nuclear genomes of haptophytes *Isochrysis galbana* and *Isochrysis* sp. was reported [223]. Subsequently, the chemical polyethylene glycol (PEG)-mediated transfer of a bacterial hygromycin B-resistance gene in a calcifying coccolithophore species *Pleurochrysis carterae* was reported [224]. Prasad reported the *Agrobacterium*-mediated nuclear transformation protocol for the metabolic engineering of *Pleurochrysis lutheri* [225]. However, CRISPR/Cas-based gene editing has not been achieved in haptophytes yet. Similarly, no CRISPR/Cas-based gene editing has been reported in dinoflagellates, another important group of phytoplankton in the ocean.

**Table 3:** CRISPR/Cas-based gene editing application on phytoplankton research.

Application	Algal Species	Target Genes	References
Nutrient regulation	*Thalassiosira pseudonana*	Urease; nitrate reductase (*NR*); silacidin; flavodoxin	[161,162,173,175]
	*Chlamydomonas reinhardtii*	Spermidine synthase (*SPD1*)	[164]
	*Phaeodactylum tricornutum*	Trypsin; alkaline phosphatase (*AP*); phosphate starvation response regulator (*PHR*); *SPX*; ferrisiderophore acquisition system (*FBP1*, *FRE1*, *FRE2*)	[165,166,168,169,172,174]
Photosynthesis and pigment biosynthesis	*Chlamydomonas reinhardtii*	Zeaxanthin epoxidase (*ZEP*); chloroplast signal recognition particle (CpSRP) receptor (*CpFTSY*)	[158,176]
	*Dunaliella salina* CCAP19/18	β-carotene hydroxylase	[186]
	*Phaeodactylum tricornutum*	Light-harvesting complex (*LHC*); chloroplast signal recognition particle 54 kDa (*CpSRP54*); CpFTSY; cryptochrome; violaxanthin de-epoxidase (*VDE*); pyruvate orthophosphate dikinase (*PPDK*); Chl *c* synthase (*CHLC*)	[178,179,180,181,182,183,184,185,188]
	*Porphyridium* sp.	Chlorophyll synthase (*CHS*)	[187]
Lipid production and fatty acid metabolism	*Chlamydomonas reinhardtii*	Phosphoenolpyruvate carboxylase (*PEPC1*); esterase lipase thioesterase (*ELT*); phospholipase A2	[194,195,196]
	*Chlorella vulgaris* FSP-E	Omega-3 fatty acid desaturase (*Fad3*)	[197]
	*Tetraselmis* sp.	ADP-glucose pyrophosphorylase (*AGP*)	[205]
	*Parachlorella kessleri*	Plastidic ATP/ADP translocase (*AATP*); duplicated mannanases 1 (*DMAN1*)	[206]
	*Phaeodactylum tricornutum*	Acyl-ACP D9-desaturase; long-chain acyl-CoA synthetases (*LACS*); monogalactosyldiacylglycerol synthase (*MGD*); stramenopile-type lipid droplet protein (*StLDP*); *NR*; glutamine synthetase 2 (*GS2*); chloroplast localized glutamate synthase(*cGOGAT*); *AP*; a novel gene *Pt2015*	[41,168,198,200,201,203,204,207,208]
Others	*Chlamydomonas reinhardtii*	Acetolactate synthase (*ALS*)	[157]
	*Volvox carteri*	*GlsA*; *regA*; *invA*	[216]
	*Picochlorum celeri*	*NR*; carotenoid isomerase	[217]
	*Ulva prolifera*	Adenine phosphoribosyl transferase (*APT*)	[218]
	*Thalassiosira pseudonana*	Putative θ-carbonic anhydrase (θ-*CA*); bestrophin-like protein (*BST2*)	[209,210]
	*Phaeodactylum tricornutum*	Metacaspase (*MCA*); single-domain voltage-gated channel (*EUKCATA*); HMP-P synthase (*THIC*); thiamine-related proteins SSSP	[212,214,215]
	*Saccharina japonica*	*APT*	[220]
	*Ectocarpus siliculosus* (*Ectocarpus* 7)	*APT*	[219]
	*Gracilariopsis lemaneiformis*	*CA*; γ subunits of phycoerythrin (*γpe*)	[221]

## 8. CRISPR/Cas in Marine Zooplankton Research

CRISPR/Cas9 technology has also increasingly been applied in zooplankton (Table 4). Effective gene knockout was successfully achieved by injecting the Cas9/sgRNA RNP complex into the egg of the hydrozoan *Clytia hemisphaerica* [226,227]. When the endogenous *GFP* genes were targeted, the fluorescence was abolished in embryos, and the functional loss of *CheRfx123* led to sperm motility defects [226]. One opsin in *C. hemiphaeria* (*Opsin9*) showed high expression in the outermost layer of astrocytes in the ovary. Using CRISPR/Cas9 technique to construct the *Opsin9*-knockout strains of *C. hemiphaeria*, the mutant strain could not release egg cells when responding to light. This indicated that *Opsin9* performs as a photoreceptor in *C. hemiphaeria* [227].

Due to high phenotypic plasticity, strong reproductive ability, small size, and important role in the aquatic food chain, water fleas have been used as an animal model species for basic biology, evolution, and ecological research. Recently, CRISPR/Cas9 technology has been successfully applied to two water flea species, *Daphnia magna* and *D. pulex*. The CRISPR/Cas9 system was employed for the knockout of the endogenous eyeless gene, which is a functionally conserved regulator of eye development in *D. magna*, resulting in heritable mutations with deformed eyes [228]. Later, a follow-up study injected Cas9 proteins and the gRNAs that target exon 10 of the eyeless gene into *D. magna* eggs, obtaining the eyeless mutants [229]. The *Dma-ey* gene in *D. magna* was also successfully mutated by the CRISPR/Cas-mediated mutagenesis, and its function in eyepoint development was revealed [228]. In another study, the distal-less gene (*DLL*), which is involved in morphological development in *D. pulex*, was knocked out by microinjecting the Cas9/dll-sgRNA RNP complex into single-cell stage embryos [230]. As a result, the second antennae and appendage of the mutant strain developed abnormally.

Serotonin plays an important role in regulating the secretion of molting and juvenile hormones in insects, and tryptophan hydroxylase (*TRH*) is the rate-limiting enzyme in the synthesis of serotonin. This CRISPR/Cas9 technology was used to create the seven indel *TRH* mutants in large fleas and revealed the physiological effects of serotonin on large fleas and its role in reproduction and growth [231]. The mutation of the *TRH* gene reduced the synthesis of serotonin, indicating that *TRH* is a key enzyme involved in the biosynthesis of serotonin, and the lack of serotonin not only reduces the growth rate and offspring size of large fleas but also the sensitivity to light. In addition to serotonin, ecdysone also plays an important role in regulating the reproduction of fleas. By using CRISPR/Cas9 technology, the mCherry reporter gene (EcRE)-controlled EcRe (*EcRE-mCherry*) that can induce ecdysone expression was inserted to generate a *EcRE-mCherry* transformant of *D. magna*, obtaining the temporal and spatial expression lineage of large fleas during embryonic development [232].

In addition, *Daphnia* typically perform parthenogenesis and only conduct sexual reproduction under unfavorable environmental conditions. Therefore, they are also a rare experimental invertebrate model for studying the mechanism of reproductive mode switch. The sex-warding mechanism in animals is related to the differences in upstream regulatory pathways of the transcription factor Doublesex (Dsx) [233]. CRISPR/Cas9 technology was used to target and abolish the transcription factor Vrille binding site in the *Dsx1* gene promoter of male embryos and caused significant downregulation of *Dsx1* [233]. This suggests that the transcription factor Vrille is responsible for activating the expression of the *Dsx1* gene in male embryos and further promoting and maintaining male shape. *Daphnia* also has long been a model for energy allocation research. A study reported a CRISPR/Cas-mediated mutation of DNA methyltransferase 3.1 (*DNMT3.1*) in *D. magna*, which could upregulate under nutrient restriction [234]. *DNMT3.1* mutant showed an increased growth rate but decreased reproduction and had a shorter lifespan under nutrient starvation. These results indicate that *DNMT3.1* acts as a key regulatory factor for longevity and energy allocation between the growth and reproduction in *D. magna* under a nutrient-limited environment.

As a prevalent group of zooplankton, rotifers have been well studied in microevolution, ecodynamics and ecotoxicology for over 100 years. Like water fleas, rotifers have a unique way of reproduction [235]. Yet the lack of gene-editing tools and transgenic strains has limited the ability to link genotypes to phenotypes and dissect molecular mechanisms. A recent study reported that CRISPR-mediated gene editing can effectively address the gap in the research [235]. *MutL* is a mismatch repair protein that plays a crucial role in meiosis in sexual organisms, and *mlh3* gene is a homologue of the *MutL* gene [235]. The knockout of *mlh3* in the rotifer *Brachionus manjavacas* resulted in the loss or reduction in males or surviving dormant eggs, a large number of undeveloped eggs and deformed structures inside the rotifer, sterile F1 offspring, and 1 or 2 small undeveloped eggs in the ovaries. In addition, most of the F1 generation died with tiny ovaries, and only two developed into similar forms to their mothers. This suggests that it is possible to use CRISPR/Cas to knock out genes in rotifers.

**Table 4:** CRISPR/Cas application in marine zooplankton research.

Species	Target Genes	References
*Clytia hemisphaerica*	*GFP*; *CheRfx123*; *Opsin9*	[226,227]
*Daphnia magna*	Eyeless; tryptophan hydroxylase (*TRH*); EcRE-mCherry; *Dsx1*; DNA methyltransferase 3.1 (*DNMT3.1*); *Dma-ey*	[228,229,231,232,233,234]
*Daphnia pulex*	Distal-less (*DLL*)	[230]
*Brachionus manjavacas*	*Mlh3*	[235]

## 9. CRISPR/Cas Application on Other Marine Animals

CRISPR/Cas technology has also been increasingly applied to research on other marine swimming animals and benthos, including Tentaculata, Hydrozoa, Anthozoa, Polychaeta, Gastropoda, Bivalvia, Crustacea, Echinoidea, Tunicata, Petromyzonti, and Teleostei (Table 5).

Coelenterates represent a basal postnatal animal from which all other postnatal animals evolved. A variety of coelenterates have been used as model organisms to perform functional research. Using CRISPR-Cas9-mediated mutagenesis, researchers found that *Notch* is essential for the normal neurogenesis and maturation of stinging cells and tentacle morphogenesis during the life stages of *Hydractinia echinata* [236]. The successful silencing of the *Brachyury* gene in lobate *Mnemiopsis leidyi* was also achieved using CRISPR/Cas9 editing. Compared with the normal development, *Bra*-Cas9-injected embryos showed consistent pharyngeal elongation defects along with a failure to extrude mesoglea (extracellular matrix, ECM) [237]. Coral reef ecosystems are of great ecological importance in the oceans. Various molecular investigations have been carried out to understand how corals respond to stress, leading to a great need for functional inquiries of specific genes and molecular pathways. Due to a lack of genetic tools for corals, this area of research has long been hindered. With the help of CRISPR/Cas technology, Cleves and colleagues successfully mutated fibroblast growth factor 1a (*FGF1a*), *GFP*, red fluorescent protein (*RFP*), and heat shock transcription factor 1 (*HSF1*) in *Acropora millepora*, demonstrating the feasibility of gene editing in stony corals [238,239]. Meanwhile, to investigate the effects of heat stress and acidification on the calcium carbonate skeletons of stony corals, CRISPR/Cas9 was used to mutate *SLC4γ* (bicarbonate transporter) in *A. millepora* juveniles. The results showed defective skeleton formation, manifesting the essential role of *SLC4γ* on skeleton formation in young coral colonies [240]. In addition, in the early-branching metazoan *Nematostella vectensis*, the native red fluorescent protein gene (*NvFP-7R*) was successfully disrupted using CRISPR/Cas9 through the use of microinjection [241]. In addition, *brachyury*, a key gene in chordate mesoderm development, is typically expressed in the pharynx precursors that divide the endoderm from the ectoderm. Using CRISPR/Cas9 gene editing, pharynx development, embryo elongation, endoderm organization, ectodermal cell polarity, and patterning along the oral–aboral axis were all impaired in *brachyury*-mutated *N. vectensis* embryos [242].

Polychaetes are the more primitive and most diverse group of annelids, the vast majority of which live in the oceans. In annelids *Capitella teleta*, one *Ct-r-opsin1* gene was knocked out using the CRISPR/Cas technology [243]. The absence of phototaxis caused by mutations in *Ct-r-opsin1* is comparable to the absence of phototaxis caused by deletion of the whole photoreceptor and pigment cell, proving that the *r-opsin* gene is essential for the phototaxis in *C. teleta*.

Mollusks are foundational fauna in the benthic community. CRISPR/Cas9-mediated transgenesis was employed to perform the mCherry fluorescent protein gene knock-in in *Crepidula fornicate* from the Lophotrochozoa superphylum, and it enables in vivo monitoring of *β*-*catenin* expression during embryonic development [244]. Another representative mollusk is the Pacific oyster (*Crassostrea gigas*). Its ability to thrive in harsh environmental conditions as a sessile filter feeder and traditional mosaic pattern of development makes it an excellent model species for ecological, evolutionary, and developmental studies. The CRISPR/Cas technology was first applied to knock out two genes, myostatin (*MSTN*) and *Twist*, in *C. gigas* [245]. A subsequent study disrupted the myosin essential light chain gene (*MELC*) in *C. gigas* larvae, and the mutant exhibited poor mobility and malformed muscles, suggesting that *MELC* functions in the myogenesis and contraction of muscles in oyster larvae [246]. In addition, Jin et al. found that the electroporation method could deliver the CRISPR/Cas9 system into the embryos of Fujian oyster *Crassostrea angulate* [247].

Gene editing studies for arthropods have also proliferated. Researchers used CRISPR/Cas9 mutagenesis to examine the function of six *Hox* genes in the crustacean amphipod *Parhyale hawaiensis*, systematically elucidating several morphological macroevolutionary shifts in the crab body facilitated by *Hox* genes [248,249]. CRISPR/Cas-based gene editing using microinjection was used in *Exopalaemon carinicauda* to knock out the chitinase gene (*EcChi4*), and the result showed that this gene is involved in immune defense [250,251]. In addition, another gene in *E. carinicauda*, molt-inhibiting hormone (*EcMIH*), was also successfully knocked out to reveal the function of this gene in suppressing the molting process [252]. In addition, the researchers went on to knock out the carotenoid isomerooxygenase (*EcNinaB-X1*) and β, β-carotene 9′, 10′-oxygenase (*EcBCO2*) genes and showed that these genes function as carotenoid isomerooxygenase in *E. carinicauda* [59,118].

Echinoderms, as deuterostome, are also the most advanced group of invertebrates, all of whom live in the oceans. It has been confirmed that *Nodal* silencing in the sea urchin *Strongylocentrotus purpuratus* using the CRISPR/Cas system can improve mutation efficiency [253]. Five of the six gRNAs created against the well-researched *nodal* gene caused the predicted phenotype in 60–80% of the injected embryos. In addition, researchers revealed that the mutation rates were 67–100% among the sequenced clones, indicating the high effectiveness of the CRISPR/Cas9 system for editing the sea urchin *S. purpuratus* [253].

Chordata has also been studied using the CRISPR/Cas editing technology. In 2014, researchers performed the first successful CRISPR/Cas-based editing of two genes, *Hox* and *Ebf*, in the sea squirt *Ciona robusta*, an ancient chordate model [254,255]. The phenotyping of transfected embryos in the ‘F0’ generation demonstrated that the specification of Islet-expressing motor ganglion neurons and atrial siphon muscles depends on endogenous *Ebf*. Subsequently, by optimizing the design of gRNAs, a CRISPR/Cas9-mediated genome editing effort successfully mutated 23 genes expressed in the cardiopharyngeal progenitors and surrounding tissues in *C. robusta* [256]. Lamprey is one of only two living jawless vertebrates. In 2015, the CRISPR/Cas system was introduced into the sea lamprey *Petromyzon marinus* to enable the modification of Tyrosinase (*Tyr*) and *FGF8/17/18* genes in the F0 generation, revealing the potential correlation between the level of albinism in a given individual and the number of mutated Tyr sequences [257]. In 2016, researchers optimized the CRISPR/Cas9 system to disrupt both alleles of all five endogenous genes in a lamprey genome, including *golden* (*gol*)*, kctd10*, *wee1*, *soxe2*, and *wnt7b* [258]. The efficient biallelic disruption produced sufficient numbers of null-phenotype and null-mutation individuals in F0, which are highly useful for genetically functional studies. More recently, Suzuki et al. reported the efficient generation of *EGFP* or *Dendra2* knock-in F0 lampreys through CRISPR-Cas9-mediated genome editing [259].

Gene editing research in fish has also developed rapidly, boosting the studies on fish gene function and the improvement of fish quality. First, zebrafish, a model species for aquatic organisms, has been proven to be amenable to CRISPR/Cas gene editing [260,261,262,263]. In addition, *solute carrier family 45 member 2* (*slc45a2*) and *tyr* gene in the F0 generation of *Salmo salar* were successfully knocked out using the CRISPR/Cas system [264]. Later, an efficient method for controlling the KI of a FLAG element in F0 salmon using CRISPR/Cas, and a symmetrical DNA repair template was developed using *slc45a2* as a gene model [265]. CRISPR-Cas technology was also used to successfully knock out the myostatin (*mstn*) gene, a negative regulator of muscle growth in red sea bream *Pagrus major* [266]. In another study, the CRISPR/Cas9-mediated knockout of the *PoMSTN* gene in olive flounder *Paralichthys olivaceus* resulted in a thickened body and increased fullness [267]. At the same time, CRISPR/Cas technology was proven to be effective in Japanese anchovy (*Engraulis japonicus*) [268]. For medaka *Oryzias melastigma,* CRISPR/Cas9 was employed to knock out *slc45a2*, which created an albino mutant phenotype [269].

**Table 5:** Applications of CRISPR/Cas in marine animal research.

Class	Species	Target Genes	References
Tentaculata	*Mnemiopsis leidyi*	*Brachyury*	[237]
Hydrozoa	*Hydractinia echinata*	*Notch*	[236]
Anthozoa	*Acropora millepora*	Fibroblast growth factor 1a (*FGF1a*); green fluorescent protein (*GFP*); red fluorescent protein (*RFP*); heat shock transcription factor 1 (*HSF1*); *SLC4γ*	[238,239,240]
	*Nematostella vectensis*	*NvFP-7R*; *brachyury*	[241,242]
Polychaeta	*Capitella teleta*	Rhabdomeric *opsin* (*Ct-r-opsin1*)	[243]
Gastropoda	*Crepidula fornicata*	*β-catenin*	[244]
Bivalvia	*Crassostrea gigas*	MSTN; *Twist*; myosin essential light chain gene (*MELC*)	[245,246]
	*Parhyale hawaiensis*	*Hox*	[248,249]
	*Exopalaemon carinicauda*	*EcChi4*; *EcMIH*; *EcNinaB-X1*; *EcBCO2*	[59,250,251,252]
Echinoidea	*Strongylocentrotus purpuratus*	*Nodal*	[253]
Tunicata	*Ciona robusta*	*Hox3*; *Hox5*; *Hox12*; *Ebf*	[254,255]
Petromyzonti	*Petromyzon marinus*	*Tyrosinase* (*Tyr*); *FGF8/17/18*; *golden* (*gol*); *kctd10*; *wee1*; *soxe2*; *wnt7b*; *LcHsp70A*	[257,258,259]
Teleostei	*Salmo salar*	*Tyr*; solute carrier family 45 member 2 (*slc45a2*)	[264,265]
	*Pagrus major*	Myostatin (*mstn*)	[266]
	*Paralichthys olivaceus*	*PoMSTN*	[267]
	*Engraulis japonicus*	Myostatin-2 (*MSTN-2*)	[268]
	*Oryzias melastigma*	*SLC45a2*	[269]

In short, gene editing has been successfully conducted in various marine animals and mostly through microinjection. However, there is still room for optimization of the current microinjection technique for some species that do not hold eggs or have fertilized eggs with fast cleavage or fragile yolks. In addition, most species manipulated so far are model species, and there is still much space for a broader application in unexplored species.

## 10. Challenges as Roadblocks

The field of the CRISPR/Cas immune system in prokaryotic microbes has flourished in the past decade. Some exciting developments have also occurred with some eukaryotes. The application of this system as a genome editing tool has also grown extremely fast. However, as technological innovation has advanced almost all branches of biology, the rapid growth of the field also has met challenges that emerged on the way. The most notable challenges include (1) low Cas enzyme efficiency and off-target editing, (2) difficulty in transforming genes in some lineages of organisms, (3) difficulty in knocking out all paralogs (isoforms) at once, and (4) difficulty in knocking out vitality/essential genes, the knockout of which would cause the death of the cell or organism.

**(1) *Low efficiency and common off-target editing*** have limited the power of the technology. To increase genome editing efficiency, researchers have strived to improve the expression of Cas or gRNA using different strategies [270]. However, the exceedingly high expression of exogenous Cas proteins can compromise the outcome. For instance, the constitutive expression of Cas genes under a strong exogenous promoter (e.g., 35S) led to cytotoxicity in *C. reinhardtii*, thereby reducing transformation efficiency [156,157]. Therefore, the screening of suitable endogenous expression elements in the CRISPR/Cas system is the ‘Golden Snitch’ [271]. Meanwhile, Cas genes should be codon-optimized according to different host species [160,272]. In addition, the CRISPR/Cas system can induce large numbers of off-target mutagenesis [273], which generates undesired mutations at random sites and upsets precise gene modification. Knockout control clones (same procedure as knockout but omitting Cas enzyme), and multiple mutant clones should be used for phenotypic analyses to discern whether off-target editing might have occurred in the procedure.

**(2) *Difficulty in transforming genes in some lineages of organisms***. Some organisms, like diatoms and green algae, are highly amenable to genetic manipulation and have served as user-friendly models for CRISPR/Cas-mediated genome editing to unlock the functions of genes. In contrast, lineages such as dinoflagellates are very calcitrant to DNA introduction and have offered little as a model in gene transformation, as the few cases of initial success [274,275,276] have yet to prove adaptable for other species.

**(3) *Challenges in knocking out all paralogs simultaneously.*** Many genes occur in multiple copies in marine organisms. For instance, there are eight alkaline phosphatase genes and ten trypsin genes in the diatom *P. tricornutum* [167,172]. Dinoflagellates are notorious for their incredibly high number of gene copies (up to 5000) [277]. In order to determine the functions of these isoforms of the enzymes or proteins, the best approach is to disrupt all these gene paralogs and reintroduce back these genes one at a time. This requires simultaneous editing of all the paralogs. Theoretically, if these multiple homologous genes share an identical functional domain that fits the sgRNA recognition framework, one editing operation may target the domain in all gene copies. However, this has been explored in some terrestrial organisms [278,279] but not yet in marine organisms.

**(4) *Frustration to edit essential vital genes.*** The loss-of-function knockout of genes essential to vitality would cause the death of the cell or organism. Some strategies have been developed to circumvent this problem [280], but it is quite tedious, and the chance of success may be variable. One alternative to gene knockout is knockdown. For instance, in a recent work by Professor Guangce Wang in collaboration with Lin on the constitutive photomorphogenesis 9 (COP9) signalosome (CSN) subunit 2 (*CSN2*) in *P. tricornutum*, CRISPR/Cas9 editing never yielded null mutants despite repeated efforts, while heterozygous mutants still expressed the intact allele of this gene but at lower levels than the wild type. This suggests that this gene is vital to this diatom. Theoretically, base substitution can be carried out to achieve different expression levels of the target gene using the CRISPR/Cas technique, but this has yet to be explored in the field of marine research.

## 11. Prospects and a Roadmap

**Figure 3:**
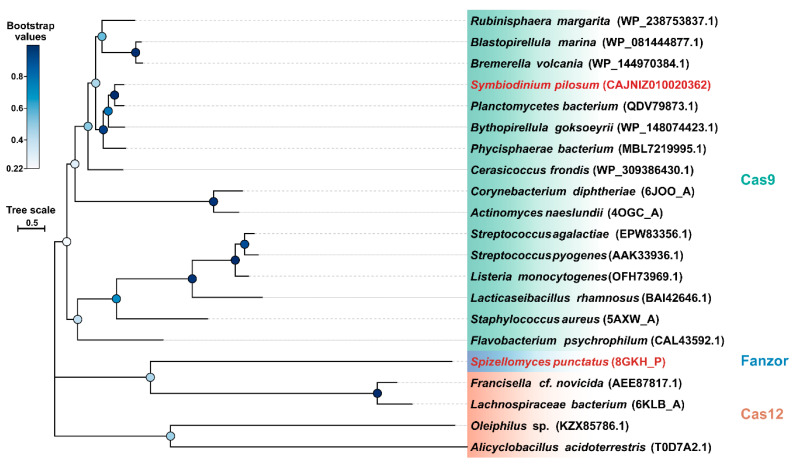
Maximum likelihood tree of the Cas9, Cas12, and Fanzor proteins from bacteria, fungus, and dinoflagellate. Color shading depicts cluster of Cas subtype named on the right. Bootstrap values on the trees were derived from 1000 resampling. In red font are eukaryotes, corresponding to Fanzor from fungi and Cas9 from dinoflagellates.

Despite the challenges, the prospect is exciting, and a roadmap is emerging. First, existing Cas enzymes can be retailored to enhance efficiency and reduce off-target edits. Various successful cases have been reported, and research in this area is ongoing. Second, new Cas enzyme systems may continue to be discovered from various organisms that may perform better than current Cas enzymes. There seems to be an enormous untapped Cas diversity within marine microorganisms, and exploring this diversity can uncover novel and previously uncharacterized CRISPR/Cas systems. CRISPR systems were initially believed to exist solely in prokaryotes, but this notion appears to be changing, as the Fanzor endonuclease-mediated system has been found in the eukaryote [281] (Figure 3). From existing algal genomes, we also detected Cas-like genes in phytoplankton. For instance, the genome of *Symbiodinium pilosum* harbors a putative Cas9 protein-coding gene that shares about 70% similarity to the Cas9 proteins in prokaryotes such as a Planctomycete (Figure 3). Whether this has been acquired through horizontal gene transfer and whether this occurs in other lineages of dinoflagellates and other groups of eukaryotic algae warrant further investigation.

**Figure 4:**
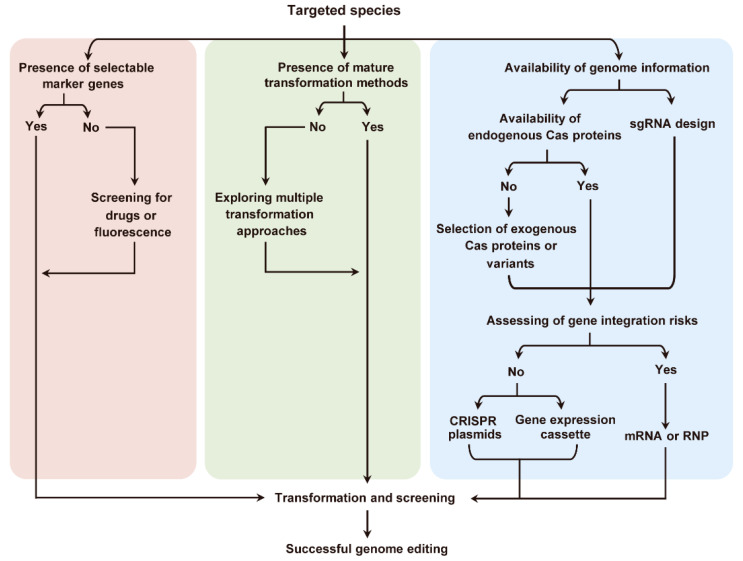
Roadmap for future applications of CRISPR/Cas in marine biological research.

Third, one of the most important inhibitory factors that limit the broad application of the technology is the difficulty in delivering the gene, RNA, or RNP protein complex into the cell. For large-sized organisms with amenable embryos, microinjection is applicable. For microorganisms like phytoplankton, the available delivery methods are restricted to electroporation, particle bombardment, or bacterial conjugation. Concerted efforts are being made in the marine protist research community to develop effective methods for DNA delivery, and successes in some lineages are providing lessons for tackling other organisms [271]. Future research should aim to integrate the achievements of CRISPR/Cas research and applications in marine organisms and develop systematic solutions, creating a comprehensive reference resource for more broadly exploiting the CRISPR/Cas technology to advance marine biology and biological oceanography. Based on the state of the art, a roadmap can be drafted, including several key considerations for success in applying CRISPR/Cas gene editing technology in marine research (Figure 4). The genome editing methodology is advancing rapidly, and it is foreseeable that many of the currently recalcitrant species (e.g., dinoflagellates) will become tractable. Notably, the methodology of using CRISPR/Cas also has been diversified. Initially, the system is introduced into cells as plasmid-based recombinant expression constructs. To date, a multi-approach is available to maximize the chance of success, including DNA, RNA, and RNA–protein complexes, as described above. More effective methods can be expected to emerge in the near future.

One interesting extension of CRISPR/Cas applications is to retailor the CRISPR/Cas system for the development of biosensors for marine ecological research. The potential has been demonstrated in a recent development of a biosensor to rapidly detect harmful algal species for monitoring purposes [282,283]. Another exciting prospect is the potential expansion of using CRISPR/Cas as a diagnostic marker for tracing the history of viral-associated microbial interactions and inferring the identification of the players. While the bacteria–virus, bacteria–bacteria–virus, or animal gut–bacteria–virus interactions have been elegantly studied as discussed earlier, the future of expanding this to microbial interactions with protists and other eukaryotes may soon emerge from the wide horizon of the vast ocean.

## Data Availability

No new data were created or analyzed in this study. Data sharing is not applicable to this article.
